# The genomic landscape of invasive stratified mucin-producing carcinoma of the uterine cervix: the first description based on whole-exome sequencing

**DOI:** 10.1186/s12967-022-03368-w

**Published:** 2022-04-25

**Authors:** Songwei Feng, Yu Ge, Xuejiao Ma, Ke Zhang, Minghui Ge, Lu Li, Yang Shen

**Affiliations:** 1 Department of Obstetrics and Gynaecology, School of Medicine, Zhongda Hospital, Southeast University, Nanjing, 210009 China; 2 State Key Laboratory of Translational Medicine and Innovative Drug Development, Jiangsu Simcere Diagnostics Co., Ltd., Nanjing, 210009 China; 3 Department of Medicine, Nanjing Simcere Medical Laboratory Science Co., Ltd., Nanjing, 210009 China

Letter to Editor,

We applaud the efforts of Farmanbar et al. for their bioinformatics research on how to use whole exome sequencing (WES) data to infer active mutational signatures in gynecological tumors [1]. The study inspired us to apply WES technology for exploring disease markers of rare diseases. In the field of gynecologic oncology, the diagnosis of rare diseases is usually made postoperatively by a senior pathologist. Even for invasive stratified mucin-producing carcinoma (ISMCs), a distinctive invasive endocervical adenocarcinoma subtype discovered by Lastra et al*.* in 2016 [2], a multi-specialist consultation may be required. ISMCs accounted for around 10% of the endocervical adenocarcinoma cases and less than 1% of the total cervical cancer cases [3]. Due to disease rarity, the clinicopathological features and genomic landscape of ISMCs remain to be unknown at present. Hence, there is an urgent need for research on the whole genomic landscape of ISMCs using WES. To lay a foundation for targeted therapy in the future, we performed a genomic analysis on 8 cases of ISMCs using WES. See Additional file 1: Additional methods for details about sample information and WES procedures.

A total of 822 single nucleotide variants (SNVs), short insertions and deletions (indels) were identified including 431 missense mutations, 206 nonsense mutations, 39 stop-lost and -gained, 43 inframe deletions and insertions, 34 frameshift deletions and insertions, as well as 69 splice region variants. *MUC4* mutated in half of the samples (4/8) (Fig. 1A), and all samples were in low TMB and microsatellite stable (MSS) status (Fig. 1B,C). In comparison of pure and mixed ISMCs, *MUC4* mutation was specific to pure ISMCs, *DMD* and *DMKN* mutations were found in most mixed ISMCs (2/3). Notably, only *MUC4* and *DMD* appeared to be the common mutated genes with high frequency in these 8 cases, compared with 49 normal adenocarcinomas in the TCGA database. The mutational spectrum of ISMCs was dominated by C>T transitions (Fig. 1D). Copy number variations (CNVs) were found in most patients (6/8), in which 4 genes (*CELA3B*, *KIR3DL2*, *UGT2B2B*, and *KIR3DL1*) were deleted and 21 genes were amplified. CNVs of *RICTOR* and *CDK4* were found in two samples. A mutational signature analysis based on the Non-Negative Matrix Factorization (NMF) algorithm was used to explore the mutational processes in ISMCs. We identified three mutational signatures in ISMCs that matched with three of the signatures in the Catalogue of Somatic Mutations in Cancer (COSMIC) database (Additional file 2: Fig. S1A). Of the three signatures, signature 1 most closely resembled COSMIC signature 1 (cosine similarity: 0.806) (Additional file 2: Fig. S1B). This signature had attributed to spontaneous deamination of 5-methycytosine. Pathway analysis showed the mutations enriched in the Wnt (4/68), Notch (4/71), receptor-tyrosine kinase (RTK)-Ras (4/85), MYC (2/13), PI3K (2/29), and Hippo (1/38) signaling pathways in ISMCs (Fig. 1E). Moreover, we also explored potential drug targets based on somatic mutational genes in DGLDB database, such as *FLT1*, *CENPF*, *DMD*, and *ADAMTS17*, etc. (Fig. 1F). Programmed death-ligand 1 (PD-L1) immunostaining (clone SP263) defined by tumor cells (TC) was conducted. Five patients were positive (TC ≥ 1%) including 4 cases of pure ISMCs and 1 case of mixed ISMCs.

**Fig. 1:**
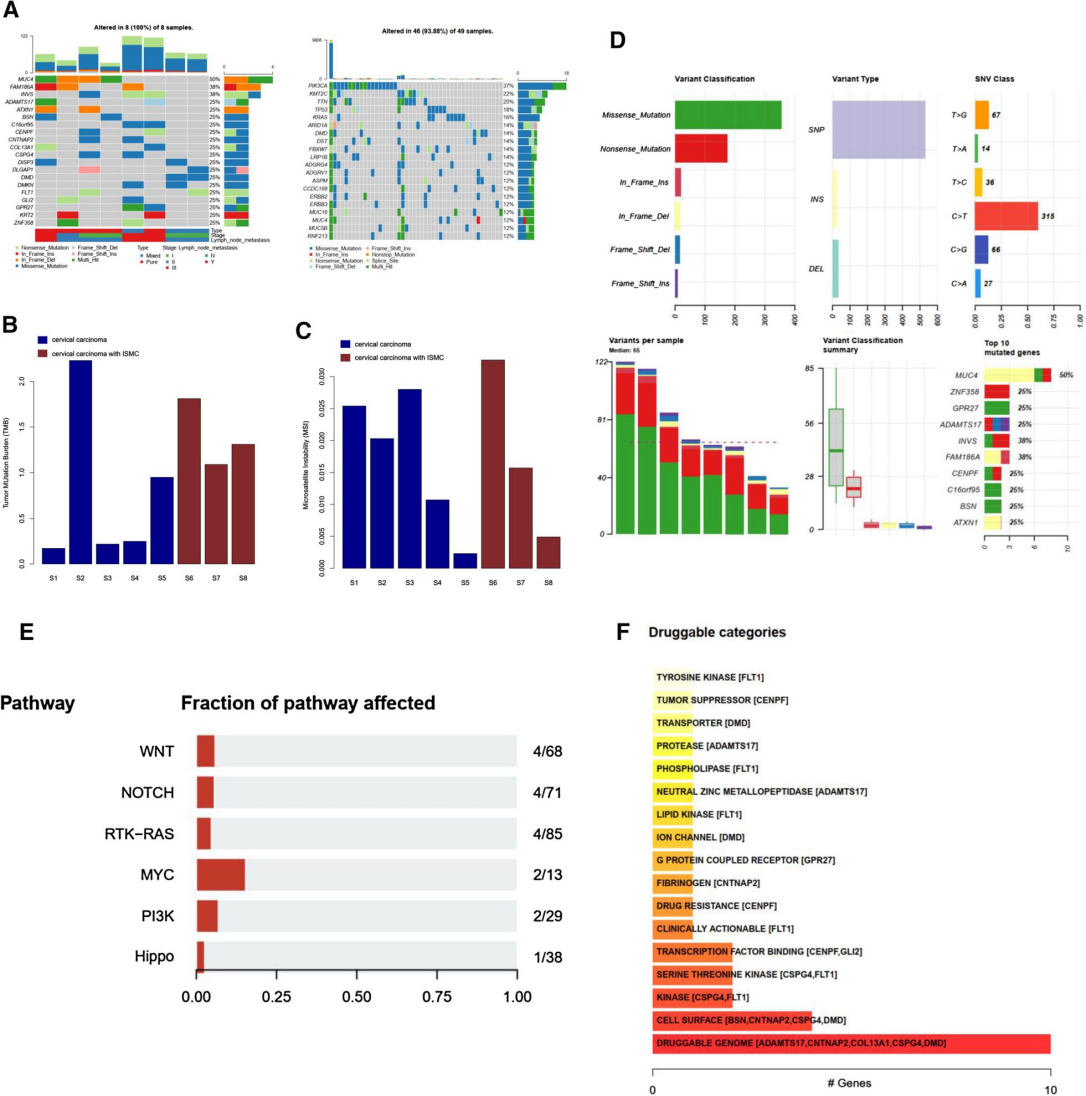
Genetic landscape of ISMCs. **A** Landscape of somatic mutations. ISMC samples on the left and TCGA adenocarcinoma samples on the right. **B**,**C** TMB and MSI scores of all samples. Blue represents pure samples and red represents mixed samples. **D** Description of variant classification, variant type, SNV class, variants per sample, variant classification summary, and top10 mutated genes. **E** Fraction of pathways affected. **F** Categories of potential drug targets

Here we described the PD-L1 expression, pathological and genetic characteristics of cervical ISMCs. Epithelial–mesenchymal transition (EMT)-related, Wnt and Notch signaling pathways were altered with a high number of genes in our research, which was consistent with EMT-prone characteristics in ISMCs reported by a previous study [4]. Further comparison between pure and mixed ISMCs revealed different molecular features in these two subtypes. *DMD* and *DMKN* mutations were predominant in mixed ISMCs. *DMD* encodes dystrophin protein (Dp) and its major products mainly expressed in skeletal muscles. However, more and more evidence showed the mutation of *DMD* and/or the altered expression of its products like Dp71 or Dp427 was associated with tumorigenesis [5]. A previous study demonstrated DMKN loss decreased tumorigenesis and could reserve EMT in pancreatic cancer cells, but the mechanism was poorly understood [6]. Overexpression of *MUC4* promoted aggressiveness and metastasis in ovarian cancer and pancreatic cancer, but in squamous cell carcinoma *MUC4* contributed to better survival. The complicated function of *MUC4* in distinct cancers and cell textures is still under investigation [7]. Additionally, MUC4 also played a pivotal role in immunosuppression by inactivation of dendritic cells and dampening the function of cytotoxic T-lymphocytes. In a preclinical study it was utilized as a target for peptide-vaccine in cancer immunotherapy [8]. Interestingly, all patients with *MUC4* mutation in our study were positive for PD-L1, suggesting these patients might benefit from MUC4-based vaccine or in combination with checkpoint inhibitors. Based on WES, we found different mutational patterns between ISMCs and normal cervical adenocarcinomas, as well as between two ISMC subtypes. *DMD*, *DMKN* and *MUC4* could be biomarkers to distinguish between pure and mixed ISMCs. Due to the small sample size and unclear mechanisms of *DMD*, *DMKN* and *MUC4* in cervical cancer, more large-scale studies need to be performed to further verify our findings and investigate the mechanism of ISMCs.

## Supplementary Information

**Additional file 1:** Additional methods.

**Additional file 2: Figure S1.** Selection of COSMIC signatures. **A** Cophenetic metric. **B** Description of matching COSMIC signatures in ISMC samples.

## Data Availability

Please contact the corresponding author for the data request.
