# Supplementary material for: The genomic landscape of invasive stratified mucin-producing carcinoma of the uterine cervix: the first description based on whole-exome sequencing
Source: J Transl Med. 2022 Apr 25;20:187. doi: 10.1186/s12967-022-03368-w (PMC9036761; doi:10.1186/s12967-022-03368-w)
Supplement: Supplementary file 1 — Additional file 1: Additional methods. [file 12967_2022_3368_MOESM1_ESM.docx]

**Additional Methods**

**Clinical Data**

Patients who underwent radical surgery for cervical cancer and had postoperative pathological confirmation of ISMCs at the Zhongda Hospital of Southeast University from 2017 to 2021 were included into this study. Clinical information included age, International Federation of Gynecology and Obstetrics (FIGO) stage, therapeutic scheme, histological grade, lymphatic vessel invasion, blood vessel invasion, and human papillomavirus (HPV) infection status. The last follow-up was conducted on February 28th, 2022, and the follow-up period was 3-58 months. No recurrences or deaths occurred in patients. All sequencing samples were taken from the surgery and only two patients underwent neoadjuvant chemotherapy preoperatively. More than half (6/8) had HPV infection at the time of diagnosis. There were 4 patients with stage IB2 tumors, three with IIIC1 tumors, and one with stage IIA2 tumor. They were at the age of 31-51 years. Five patients were diagnosed as pure ISMCs, and the remaining three were diagnosed as mixed ISMCs, including co-existing usual type of endocervical adenocarcinoma (UAE), co-existing squamous cell carcinoma (SCC), as well as adenocarcinoma in situ (AIS), high-grade squamous intraepithelial lesion (HSIL) and stratified mucin-producing intraepithelial lesion (SMILE). Lymph node metastases were detected in three cases. Vessel metastases were detected in four cases. See the **Table** below for more details.

**Table. Clinicopathological features of 8 ISMC patients**

| Cases | Age | Pathologic types | HPV types | FIGO stage | NACT | LVI | BVI |
| --- | --- | --- | --- | --- | --- | --- | --- |
| 1 | 49 | Pure ISMCs | 18 | IB2 | No | No | No |
| 2 | 47 | Pure ISMCs | / | IIIC1 | Yes | Yes | Yes |
| 3 | 43 | Pure ISMCs | 16 | IB2 | No | No | Yes |
| 4 | 48 | Pure ISMCs | 18 | IIA2 | Yes | No | Yes |
| 5 | 31 | Mixed ISMCs (UAE) | / | IIIC1 | No | Yes | No |
| 6 | 51 | Mixed ISMCs (AIS, HSIL, SMILE) | 18 | IB2 | Yes | No | No |
| 7 | 36 | Pure ISMCs | 18 | IIIC1 | No | Yes | Yes |
| 8 | 44 | Mixed ISMCs (SCC) | / | IB2 | No | No | No |

ISMC, invasive stratified mucin-producing carcinoma; UAE, endocervical adenocarcinoma; AIS, adenocarcinoma in situ; HSIL, high-grade squamous intraepithelial lesion; SMILE, stratified mucin-producing intraepithelial lesion; SCC, squamous cell carcinoma; NACT, new adjuvant chemotherapy treatment; LVI, lymphatic vessel invasion; BVI, blood vessel invasion; FIGO, International Federation of Gynecology and Obstetrics; HPV, human papillomavirus.

**Whole exome sequencing**

Genomic DNA was extracted from formalin-fixed paraffin-embedded (FFPE) samples with more than 20% tumor cells and from matched blood following manufacturer’s protocol. Library construction was performed using the KAPA Library Preparation kit. The concentration of the library was assessed using the Invitrogen Qubit4.0, and the inserted size was examined on the Agilent 4200 TapeStation. Sequencing was performed on the Illumina Novaseq 6000 system at an average depth of 500X with whole exome. A custom pipeline was built including reads alignment, variants calling, identification of fusion and copy number variations (CNVs), quality control. The fastp (v.2.20.0) was performed for adapter-trimming. Cleaned paired-end reads in FASTQ format were aligned to the human reference genome (hg19) with BWA-mem (v.0.7.17) and selected regions were realigned with ABRA2(v2.21). VarDict (v.1.5.7) [1] and InterVar [2] were utilized to call both single nucleotide variants (SNVs) and short insertion and deletion variants (indels). CNVs and fusions were identified by CNVkit (dx1.1) [3] and FACTERA (v1.4.4) [4], respectively. Additional analysis of mutation filter and inspection was implemented by custom scripts.

1. Lai Z, Markovets A, Ahdesmaki M, et al. VarDict: a novel and versatile variant caller for next-generation sequencing in cancer research. Nucleic Acids Res. 2016;44(11):e108. doi:10.1093/nar/gkw227
2. Li Q, Wang K. InterVar: Clinical Interpretation of Genetic Variants by the 2015 ACMG-AMP Guidelines. Am J Hum Genet. 2017;100(2):267-280. doi:10.1016/j.ajhg.2017.01.004
3. Talevich E, Shain AH, Botton T, Bastian BC. CNVkit: Genome-Wide Copy Number Detection and Visualization from Targeted DNA Sequencing. PLoS Comput Biol. 2016;12(4):e1004873. Published 2016 Apr 21. doi:10.1371/journal.pcbi.1004873
4. Newman AM, Bratman SV, Stehr H, et al. FACTERA: a practical method for the discovery of genomic rearrangements at breakpoint resolution. Bioinformatics. 2014;30(23):3390-3393. doi:10.1093/bioinformatics/btu549
