# Supplementary figures and images for: The genomic landscape of invasive stratified mucin-producing carcinoma of the uterine cervix: the first description based on whole-exome sequencing
Source: J Transl Med. 2022 Apr 25;20:187. doi: 10.1186/s12967-022-03368-w (PMC9036761; doi:10.1186/s12967-022-03368-w)

**A***cophenetic metric*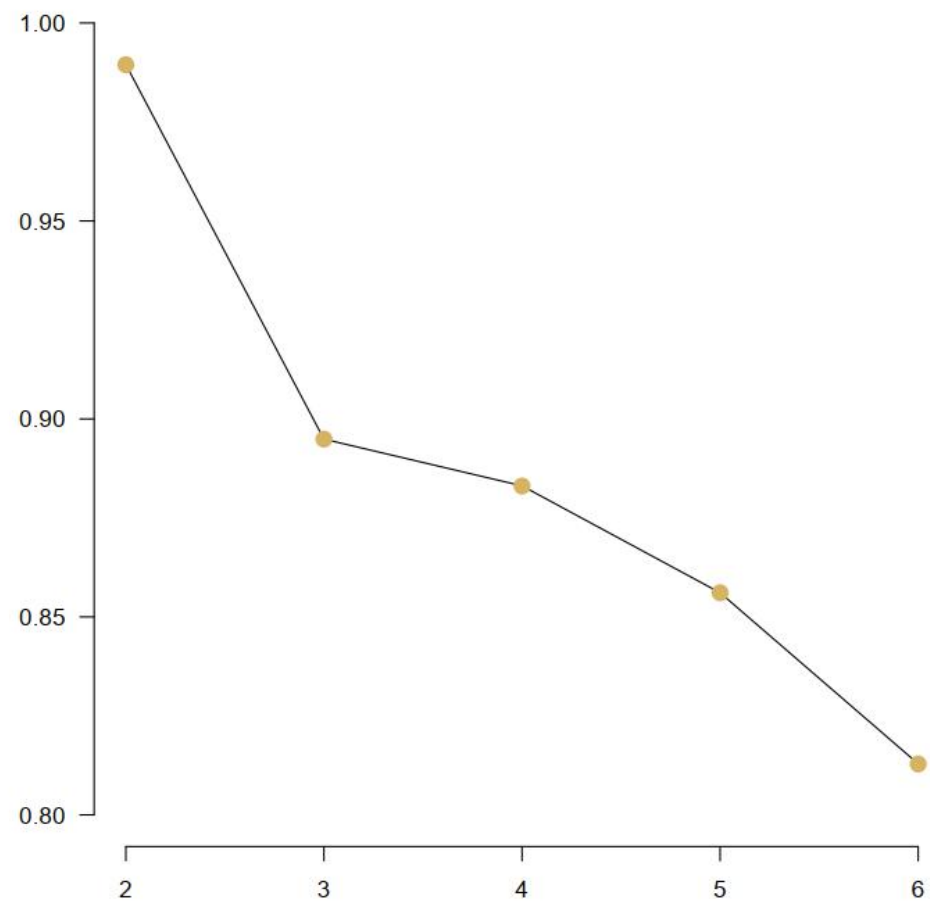**B**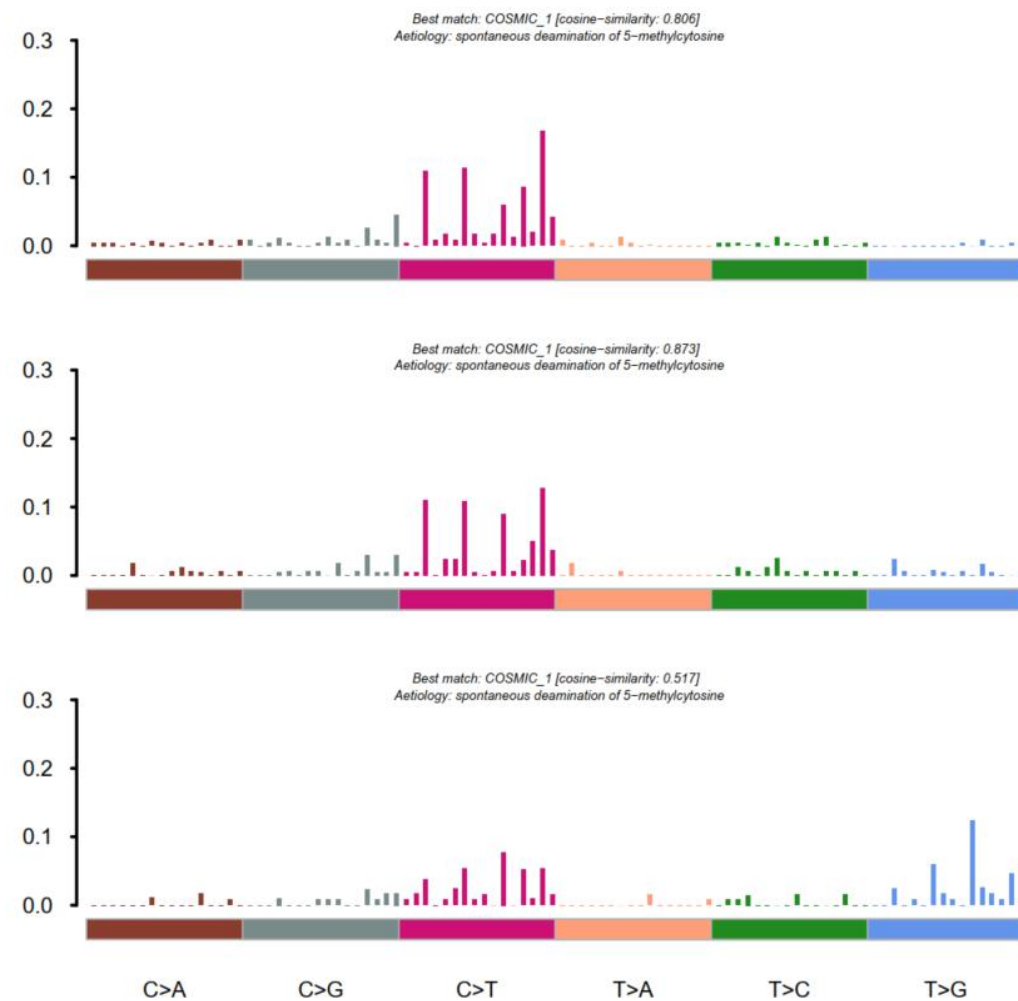

Supplement: Supplementary file 2 — Additional file 2: Figure S1. Selection of COSMIC signatures. A Cophenetic metric. B Description of matching COSMIC signatures in ISMC samples. [file 12967_2022_3368_MOESM2_ESM.pdf]
